# Novel Peptide-Modified Zeolitic Imidazolate Framework-8 Nanoparticles with pH-Sensitive Release of Doxorubicin for Targeted Treatment of Colorectal Cancer

**DOI:** 10.3390/pharmaceutics17020246

**Published:** 2025-02-13

**Authors:** Liming Gong, Heming Zhao, Liqing Chen, Yanhong Liu, Hao Wu, Chao Liu, Jing Feng, Chenfei Liu, Congcong Xiao, Qiming Wang, Mingji Jin, Zhonggao Gao, Wei Huang, Youyan Guan

**Affiliations:** 1 State Key Laboratory of Bioactive Substance and Function of Natural Medicines, Institute of Materia Medica, Chinese Academy of Medical Sciences and Peking Union Medical College, Beijing 100050, China; dawngong@163.com (L.G.); heming.zhao@btyy.com (H.Z.); chenliqing@imm.ac.cn (L.C.); liuyanhong@imm.ac.cn (Y.L.); wuhao931230@163.com (H.W.); liuchao@bme.pumc.edu.cn (C.L.); fengjinga@imm.ac.cn (J.F.); liuchenfei@imm.ac.cn (C.L.); xiaocongcong@imm.ac.cn (C.X.); wqmxinyou@imm.ac.cn (Q.W.); jinmingji@imm.ac.cn (M.J.); zggao@imm.ac.cn (Z.G.); 2 Beijing Key Laboratory of Drug Delivery Technology and Novel Formulations, Department of Pharmaceutics, Institute of Materia Medica, Chinese Academy of Medical Sciences and Peking Union Medical College, Beijing 100050, China; 3 Department of Urology, National Cancer Center, National Clinical Research Center for Cancer, Cancer Hospital, Chinese Academy of Medical Sciences and Peking Union Medical College, Beijing 100021, China

**Keywords:** LT peptide, zeolitic imidazolate framework-8, pH-sensitive release, doxorubicin, colorectal cancer, targeted tumor therapy

## Abstract

**Background**: Colorectal cancer (CRC) is one of the common malignant tumors. Chemotherapeutic agents represented by doxorubicin (DOX) are common adjuvant therapies for patients with advanced CRC. However, DOX suffers from dose-dependent cardiotoxicity and myelosuppression due to a lack of targeting and specificity, which severely limits its clinical application. **Methods**: Herein, we constructed a zeolitic imidazolate framework-8 (ZIF-8) modified by a novel peptide (LT peptide) to deliver the chemotherapeutic drug doxorubicin (DOX) for the targeted treatment of CRC. **Results**: In this study, LT-PEG@DOX@ZIF-8 nanoparticles were prepared by a simple method with suitable particle size and zeta potential, which were also capable of pH-responsive drug release. In vitro assays exhibited that LT-PEG@DOX@ZIF-8 nanoparticles were effectively taken up by C26 cells, significantly inhibited cell proliferation, and induced apoptosis. Furthermore, in mice models with colorectal tumors, LT-PEG@DOX@ZIF-8 nanoparticles also displayed specific tumor aggregation and exerted anti-tumor effects to prolong the survival of the mice. **Conclusions**: In conclusion, LT-PEG@DOX@ZIF-8 provides a promising strategy for the delivery of DOX to effectively treat CRC.

## 1. Introduction

Colorectal cancer (CRC) is the third most common malignant tumor in the world, occurring mostly in middle-aged and elderly men aged 41–60 years. The proportion of rectal cancers is higher than that of CRC, accounting for about 60% of cases [1]. By 2030, the incidence of colon and rectal cancers diagnosed in the adult population is expected to be 11% and 23%, respectively [2]. Due to the characteristics of CRC with extensive localized lesions and adhesions, complete resection cannot be achieved by surgery, thus presenting a risk of recurrence and metastasis [3]. Chemotherapy is the common adjuvant treatment for CRC patients, especially those with advanced disease [4,5]. As a wide-spectrum anti-tumor agent, doxorubicin (DOX) is effective against a variety of tumors, including colorectal tumors [6]. However, DOX suffers from dose-dependent cardiotoxicity and myelosuppression, which greatly limits its clinical application [7,8]. To address the application limitations of DOX, extensive studies have focused on targeting delivery systems to improve tissue selectivity and reduce systemic toxicity [9].

Metal–organic frameworks (MOFs) are a novel class of porous organic coordination frameworks that have attracted growing attention in the fields of biomedical, chemical, and material sciences, with wide applications in the areas of adsorption, catalysis, sensing and gas storage, as well as drug delivery [10,11,12,13,14,15]. In the case of the application of MOFs as drug delivery systems, biocompatibility is a crucial issue. Hence, the toxicity of metal ions and organic ligands in MOFs must be considered. As an endogenous metal ion, the Zn^2+^ ion is an essential trace element for humans that is widely applied in the construction of MOFs. Zeolitic imidazolate framework-8 (ZIF-8), a subclass of MOFs derived from the self-assembled binding of Zn^2+^ ions to 2-methylimidazole (2-MIM), has been broadly adopted in drug delivery systems due to its superior biocompatibility [16,17,18]. It has been reported that ZIF-8 has the unique advantages of controlled synthesis, good chemical stability, and a high encapsulation rate [19,20,21,22]. Moreover, ZIF-8 features pH-responsive properties and thus can remain stable in the blood circulation while degrading and releasing drugs in the acidic environment of endosomes/lysosomes of tumors [23,24,25]. Therefore, ZIF-8 deserves to be thoroughly investigated as a promising drug delivery system.

Ligand-mediated diagnostics and targeted therapies have important potential for clinical applications in cancer treatment, among which peptide ligands have attracted widespread attention. The cyclic peptide TCP-1 (c[CTPSPFSHC]OH) was initially discovered in orthotopic CRC mice by phage display selection [26,27]. Subsequent studies have shown that TCP-1 can target CRC tissue to enable cancer diagnosis and treatment [28]. Based on TCP-1, we simplified the structure of the peptide and designed the LT peptide (TPSPFSHC), aiming to target colorectal tumors. Thus, LT peptides are employed as peptide ligands to construct targeted delivery systems.

**Scheme 1:**
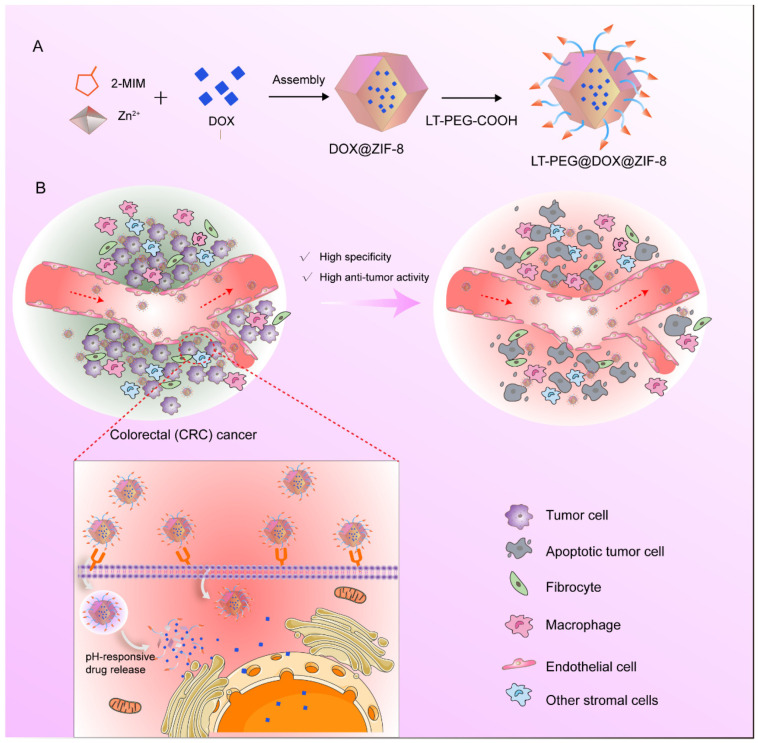
Schematic diagram of the preparation and therapeutic mechanism of LT-PEG@DOX@ZIF-8. (**A**) The preparation of LT-PEG@DOX@ZIF-8. (**B**) Schematic illustration of LT-PEG@DOX@ZIF-8 NPs specifically accumulating and exerting anti-tumor efficacy in tumor tissues.

In this study, we constructed LT-PEG@DOX@ZIF-8 nanoparticles to deliver the chemotherapeutic drug DOX for the treatment of colorectal tumors, which were obtained by modifying LT-PEG-COOH on the DOX@ZIF-8 surface through electrostatic interactions (Scheme 1). Firstly, we investigated the physicochemical properties of LT-PEG@DOX@ZIF-8 nanoparticles, including average particle size, surface potential, storage stability, and pH-responsive release behavior. Subsequently, in vitro cellular experiments were performed to evaluate LT-PEG@DOX@ZIF-8 nanoparticles for cellular uptake, proliferation inhibition, and induction of apoptosis in colorectal tumor (C26) cells. Finally, we studied the distribution of LT-PEG@DOX@ZIF-8 nanoparticles in vivo, investigated the effect on tumor ablation, and conducted a safety evaluation in mice models with orthotopic colorectal tumors. It could be concluded that LT-PEG@DOX@ZIF-8 nanoparticles may have great potential to treat CRC.

## 2. Materials and Methods

### 2.1. Materials

Doxorubicin was purchased from Shanghai Aladdin Biochemical Technology Co., Ltd. (Shanghai, China). The peptide LT (TPSPFSHC) was synthesized by Tanshui-Tech (Guangzhou, China). Methoxy-(polyethylene glycol)_2000_-COOH (mPEG_2000_-COOH) and Maleimide-(polyethylene glycol)_2000_-COOH (Mal-PEG_2000_-COOH) were bought from AVT Shanghai Pharmaceutical Tech Co., Ltd. (Shanghai, China). Zinc nitrate hexahydrate was commercially obtained from the Lanzhou Yellow River Institute of Zinc and Magnesium Nanomaterials (Lanzhou, China). 2-Methylimidazole (2-MIM) was bought from Beijing Huabozhan Bioanalytical Technology Co., Ltd. (Beijing, China). Dimethyl sulfoxide and coumarin 6 (Cou-6) were purchased from Sigma-Aldrich Co. (St. Louis, MO, USA).

PBS (Phosphate Buffer Saline), FBS (Fetal Bovine Serum), and RPMI-1640 medium were bought from GIBCO, Invitrogen Corp. (Carlsbad, CA, USA). Cell-Counting Kit-8 (CCK-8) was obtained from Dojindo Laboratories (Kumamoto, Japan). DAPI and Hoechst 33258 were bought from Shanghai Beyotime Co., Ltd. (Shanghai, China). The fluorescein isothiocyanate (FITC)-Annexin V/propidium iodide (PI) apoptosis detection kit was purchased from Solarbio Life Sciences Co., Ltd. (Beijing, China).

### 2.2. Synthesis and Characterization of LT-PEG-COOH

As previously reported, LT-PEG-COOH was synthesized by linking the cysteine residue of LT to Mal-PEG-COOH [29]. In brief, Mal-PEG-COOH and LT peptide were dissolved in HEPES buffer (pH 8.0) at the molar ratio of 1:1.2. The mixture was stirred and reacted at room temperature in nitrogen for 18 h. The final reaction mixture was dialyzed for 6 h in a MWCO 1000 Da dialysis bag to remove the free LT. The pure product was obtained via cryodesiccation. ^1^H nuclear magnetic resonance (NMR) spectroscopy (500 MHz, Varian Medical Systems, Inc., Palo Alto, CA, USA) was employed to confirm the structure of LT-PEG-COOH dissolved in D_2_O at 25 °C (acquisition conditions described in the Supporting Information). Matrix-assisted laser desorption–ionization time-of-flight mass spectrometry (MALDI-TOF-MS, 4800 Plus, Applied Biosystems Inc., Waltham, MA, USA) was utilized to further analyze the molecular weights of LT, Mal-PEG-COOH, and LT-PEG-COOH with 3-indoleacetic acid (IAA) as the detection matrix.

### 2.3. Preparation of LT-PEG@DOX@ZIF-8

The one-pot method was applied to prepare DOX@ZIF-8 nanoparticles according to the previous literature [30]. In detail, 15.2 mg DOX was dissolved in 1.52 mL of NaOH (pH 8.0) solution. Then, the DOX solution was added to dimethyl sulfoxide with 80 mg of zinc nitrate hexahydrate, fully dissolved, and stirred for 0.5 h. Finally, 800 mg of 2-MIM, adequately dissolved in water, was added drop by drop to the above mixture and stirred for 0.5 h. After the reaction, the product was collected by centrifugation, followed by washing three times with methanol. Then, DOX@ZIF-8 nanoparticles were obtained by lyophilization.

LT-PEG@DOX@ZIF-8 nanoparticles were obtained via electrostatic interaction to coat LT-PEG-COOH on their surfaces. LT-PEG-COOH and DOX@ZIF-8 were dissolved in deionized water (mass ratio: LT-PEG-COOH: DOX@ZIF-8 = 1:10) and incubated for 12 h with stirring. LT-PEG@DOX@ZIF-8 NPs were obtained by centrifugation.

### 2.4. Characterization of LT-PEG@DOX@ZIF-8

Dynamic light scattering (DLS) and electrophoretic light scattering were adopted to determine the mean particle size, polydispersity index (PDI), and zeta potential of LT-PEG@PTX@ZIF-8. The morphology of LT-PEG@PTX@ZIF-8 was characterized by transmission electron microscopy (TEM).

The encapsulation efficiency (EE%) and drug loading capacity (DL%) were determined using high-performance liquid chromatography (HPLC). The HPLC chromatographic conditions were as follows: column, Agilent-C18 (5 μm, 250 mm × 4.6 mm, Agilent Technologies, Santa Clara, CA, USA); detection wavelength, 254 nm; column temperature, 25 °C; mobile phase, sodium dodecyl sulfate solution (containing 1.44 g of sodium dodecyl sulfate and 0.68 mL of phosphoric acid)–acetonitrile–methanol (500:500:60); and flow rate, 1 mL/min.

A total of 1 mL of the LT-PEG@DOX@ZIF-8 nanoparticle solution was centrifuged to collect the supernatant, which was diluted to 10 mL with acetonitrile to determine the DOX content by HPLC (W_supernatant_). A total of 1 mL of the LT-PEG@DOX@ZIF-8 nanoparticle solution was completely destroyed by adding a hydrochloric acid solution and then diluted to 10 mL with acetonitrile to determine the DOX content (W_total drug_). A total of 1 mL of the LT-PEG@DOX@ZIF-8 nanoparticle solution was freeze-dried and weighed (W_total nanoparticles_). The EE% and DL% of DOX were calculated as follows:

$$EE\%=\frac{W_{\mathrm{loaded}}}{W_{\mathrm{total}\;\mathrm{drug}}}\times 100\%$$

$$DL\%=\frac{W_{\mathrm{loaded}}}{W_{\mathrm{total}\;\mathrm{nanoparticles}}}\times 100\%$$

where W_loaded_ = the weight of DOX encapsulated in the nanoparticles (W_total drug_ − W_supernatant_), W_total drug_ = total weight of DOX added, and W_total nanoparticles_ = the weight of nanoparticles.

### 2.5. Stability of LT-PEG@DOX@ZIF-8

To investigate the stability of the nanoparticles, the Zetasizer Nano ZS90 instrument (Malvern Instruments Ltd., Malvern, UK) was employed to measure the particle size and PDI of LT-PEG@DOX@ZIF-8 NPs. Subsequently, they were incubated in PBS (pH 7.4) at 4 °C, and the particle sizes were measured once a day for 6 days.

### 2.6. In Vitro Release Assay

Dialysis was conducted to investigate the release properties of LT-PEG@DOX@ZIF-8. In detail, dialysis bags (MWCO 1000D, Biosharp, Beijing, China) containing 1 mL of the LT-PEG@DOX@ZIF-8 solution were placed in 20 mL of PBS solution at pH 7.4 and pH 5.5, respectively. The samples were shaken at a rotational speed of 100 r/min at 37 °C. At the programmed time point within 48 h, 0.5 mL of the solution was removed, and the corresponding release medium was replenished. The media were centrifuged to separate the supernatant. The drug concentration was determined by HPLC to calculate the cumulative drug release.

### 2.7. Cell Culture

Mouse colorectal cancer cells (C26 cells) were acquired from the Cell Culture Center of the Institute of Basic Medical Sciences at the Chinese Academy of Medical Sciences. The C26 cells were cultured in 1640 medium supplemented with 10% FBS, penicillin (100 units/mL), and streptomycin (100 μg/mL) in a 5% CO_2_ atmosphere at 37 °C. Cellular experiments were performed on the logarithmically growing cells.

### 2.8. Cellular Uptake Studies In Vitro

To more visually observe the uptake of nanoparticles by C26 cells in vitro, coumarin-6 (Cou-6) was encapsulated in the nanoparticles. C26 cells were seeded in a 12-well plate (1 × 10^5^/well) and incubated for 24 h. Then, the serum-free RPMI-1640 medium diluted with free Cou-6, mPEG@Cou-6@ZIF-8, and LT-PEG@Cou-6@ZIF-8 (Cou-6, 1 μg/mL) were added. After continuing to incubate for 1 h, 1.5 h, and 2 h, the cells were fixed with 4% paraformaldehyde, and the nuclei were stained with DAPI. The cellular uptake in the C26 cells at different culture times was observed under confocal laser scanning microscopy (CLSM).

Flow cytometry was employed to investigate the cellular uptake efficiency quantitatively. C26 cells were cultured in 6-well plates (1 × 10^5^/well); then, the serum-free RPMI-1640 medium containing free Cou-6, mPEG@Cou-6@ZIF-8, and LT-PEG@Cou-6@ZIF-8 (Cou-6, 1 μg/mL) were added to each well. After continuing to incubate for 1 h, 1.5 h, and 2 h, the cells were collected and resuspended with PBS. Cellular uptake was assayed and screened for appropriate timing to compare the targeting properties of the different preparations using flow cytometry.

### 2.9. Cell Viability Assay

The inhibition of cell proliferation by different preparations on C26 cells was evaluated using CCK-8 assays. In brief, C26 cells were seeded in 96-well plates (5000/well) and incubated for 24 h. Then, different concentrations of various preparations were added. After incubation for 48 h, serum-free medium containing 10% CCK-8 was added to each well under light-avoiding conditions, and then the absorbance value at 450 nm of each well was measured by a Synergy H1 Microplate Reader. Cell viability was calculated according to the following formula:

$$\mathrm{Cell}\;\mathrm{viability}\left(\%\right)=\frac{{OD}_{test}-{OD}_{blank}}{{OD}_{control}-{OD}_{blank}}\times 100\%$$

The effect of blank ZIF-8 at various concentrations on C26 cell viability was also investigated by the CCK-8 assay following a similar procedure as described above.

### 2.10. Cell Apoptosis Assay

The cell apoptosis of C26 cells treated with different preparations was analyzed by Hoechst staining. C26 cells were seeded in 12-well plates (1 × 10^5^/well) and incubated for 24 h. Then, various preparations (containing DOX: 0.5 µg/mL) were added, followed by continued incubation for 48 h. After being fixed with 4% paraformaldehyde and stained with Hoechst 33258 (5 μg/mL), the C26 cells were observed under an inverted fluorescence microscope to analyze the morphological changes in their nuclei.

The Annexin V-FITC/PI double staining assay was applied to quantitatively analyze the apoptosis of C26 cells. Similar to above, C26 cells were inoculated in 6-well plates (1 × 10^5^ cells/well) and incubated for 24 h. Then, various preparations (containing DOX: 0.5 µg/mL) were added, followed by continued incubation for 48 h. The cells were collected and resuspended with a binding buffer and then stained with Annexin V-FITC and PI. The DOX-treated cells were mono-stained with Annexin V-FITC and PI as the conditions for adjusting compensation. The percentage of apoptosis cells was quantitatively analyzed using a flow cytometer.

### 2.11. In Vitro Wound-Healing Assay

To explore the effect of nanoparticles on the migration and mobility of C26 cells, wound-healing assays were performed for various preparations. After seeding C26 cells into a 6-well plate and incubating for 24 h, a wound was scratched in each well, and the floating cells were rinsed. Then, various preparations (containing DOX: 0.5 µg/mL) were incorporated into different wells. The shape of the wound area was recorded using an inverted light microscope after 0, 12, 24, and 48 h.

### 2.12. Establishment of CRC Model

C26 cells in the logarithmic growth phase were injected into the rectum wall of BALB/c mice to construct mouse models with CRC. Briefly, male BALB/c mice were anesthetized with chloral hydrate, and their anuses were flaccidly opened with forceps. An insulin needle was loaded with 100 µL cell suspension (containing 5 × 10^5^ cells) and injected deep into each anus into the mucosal layer of the rectum wall. C26-bearing mice were kept for 7 days and used for subsequent experiments.

### 2.13. Biodistribution and In Vivo Imaging

The biodistribution of nanoparticles injected systemically was explored by an in vivo imaging system (Caliper Life Sciences Inc., Mountain View, CA, USA). New indocyanine green (IR-820) was chosen to replace DOX for encapsulation in ZIF-8 to be easily observed in vivo. BALB/c mice bearing colorectal tumor cells were randomly divided into 3 groups, which were given various preparations by intravenous injection through the tail vein (100 µL, 2 mg/kg of IR-820). The fluorescence signal of IR-820 was detected by an in vivo imaging system at 1, 2, 4, 6, 8, and 24 h of administration. Subsequently, the mice were euthanized, and their major organs (tumor, heart, liver, spleen, lungs, and kidneys) were dissected to further analyze the distribution of nanoparticles in vivo. The fluorescence signals of the major organs were recorded using an in vivo imaging system, and Living Image software (version 4.3.1; Caliper Life Sciences Inc.) was employed to quantify the fluorescence signal intensity.

### 2.14. In Vivo Colorectal Tumor Cell Distribution

C26-bearing BALB/c mice were randomly grouped and injected with saline, LT-PEG@ZIF-8, free DOX, mPEG@DOX@ZIF-8, and LT-PEG@DOX@ZIF-8 through the tail vein (100 µL, 1.5 mg/kg of DOX). The mice were euthanized, and tumor tissues were immediately processed by frozen section after 4 h of drug administration. The fluorescence distribution of nanoparticles in the tumor tissue was observed under CLSM.

### 2.15. In Vivo Anti-Tumor Efficiency and Biosafety Evaluation

C26-bearing BALB/c mice were divided randomly into 5 groups, which included saline, LT-PEG@ZIF-8, free DOX, mPEG@DOX@ZIF-8, and LT-PEG@DOX@ZIF-8 groups. The mice were given different preparations administered by tail vein injection every 3 days for 5 times (100 µL, DOX: 2.5 mg/kg). The body weight of each mouse was recorded during treatments. All the mice were euthanized, and their major organs (including heart, liver, spleen, lung, and kidney) and tumors were dissected on day 22. The major organs were stained with hematoxylin and eosin (HE) to investigate the antimetastatic efficacy and analyze biocompatibility and tissue cytotoxicity. The tumor tissues were weighed and sliced for terminal deoxynucleotidyltransferase-mediated nick end labeling (TUNEL) staining to detect cell apoptosis. Finally, the blood of the mice was analyzed with the specific tests of alanine aminotransferase (ALT), blood urea nitrogen (BUN), azelaic aminotransferase (AST), and creatinine (CRE).

### 2.16. Statistical Analysis

All experimental data are presented as mean ± standard deviation (SD). The data were analyzed statistically using Student’s *t*-test or one-way ANOVA by GraphPad Prism software version 8. Significant differences between experimental groups were set as * *p* < 0.05, ** *p* < 0.01, and *** *p* < 0.001.

## 3. Results

### 3.1. Synthesis of LT-PEG-COOH

**Figure 1:**
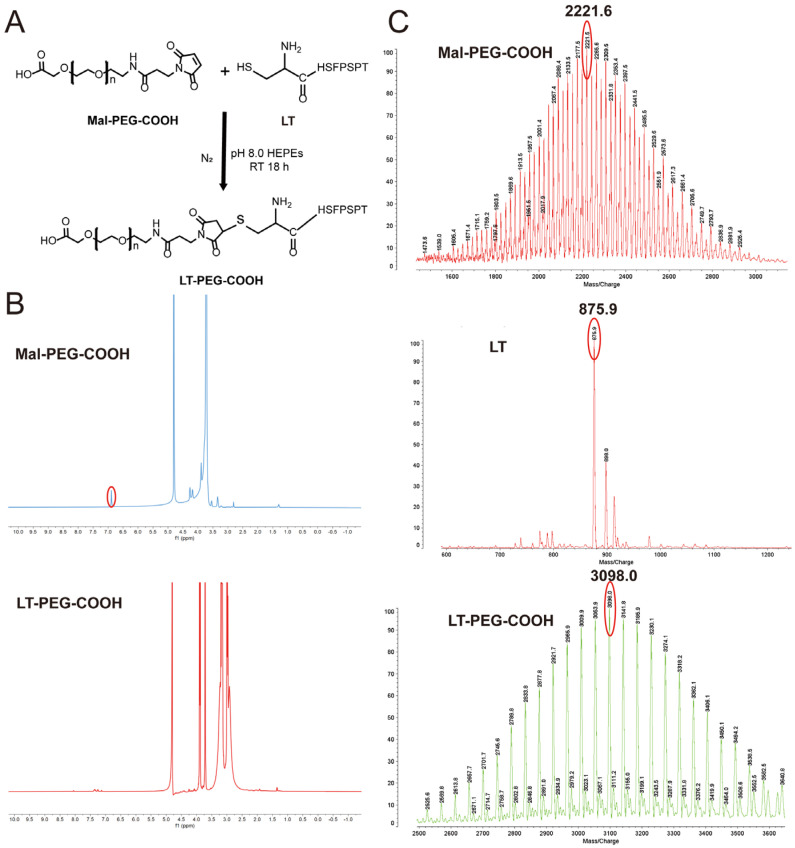
Synthesis of LT-PEG-COOH. (**A**) Synthesis route of LT-PEG-COOH. (**B**) ^1^H NMR spectra of Mal-PEG-COOH and LT-PEG-COOH (D_2_O: 4.79 ppm). (**C**) MALDI-TOF-MS spectra of Mal-PEG-COOH, LT, and LT-PEG-COOH.

As shown in Figure 1A, LT-PEG-COOH was obtained by combining the sulfhydryl group of the cysteine at the terminus of the LT peptide with the maleimide group of Mal-PEG-COOH. To verify the successful synthesis of LT-PEG-COOH, ^1^H NMR was performed to corroborate its structure. The absorption peak at 6.8 ppm is the characteristic absorption peak of the maleimide group, which is present in the ^1^H NMR spectrum of Mal-PEG-COOH but disappears in the spectrum of LT-PEG-COOH, suggesting successful synthesis (Figure 1B). Moreover, as can be seen in Figure 1C, MALDI-TOF-MS showed that the average molecular weights of Mal-PEG-COOH, LT, and LT-PEG-COOH were 2221.6, 875.9, and 3098 Da, respectively, which were, in general, consistent with their theoretical values. Thus, both structural characteristics and molecular weight can prove the successful synthesis of LT-PEG-COOH.

### 3.2. Characterization of LT-PEG@DOX@ZIF-8

**Figure 2:**
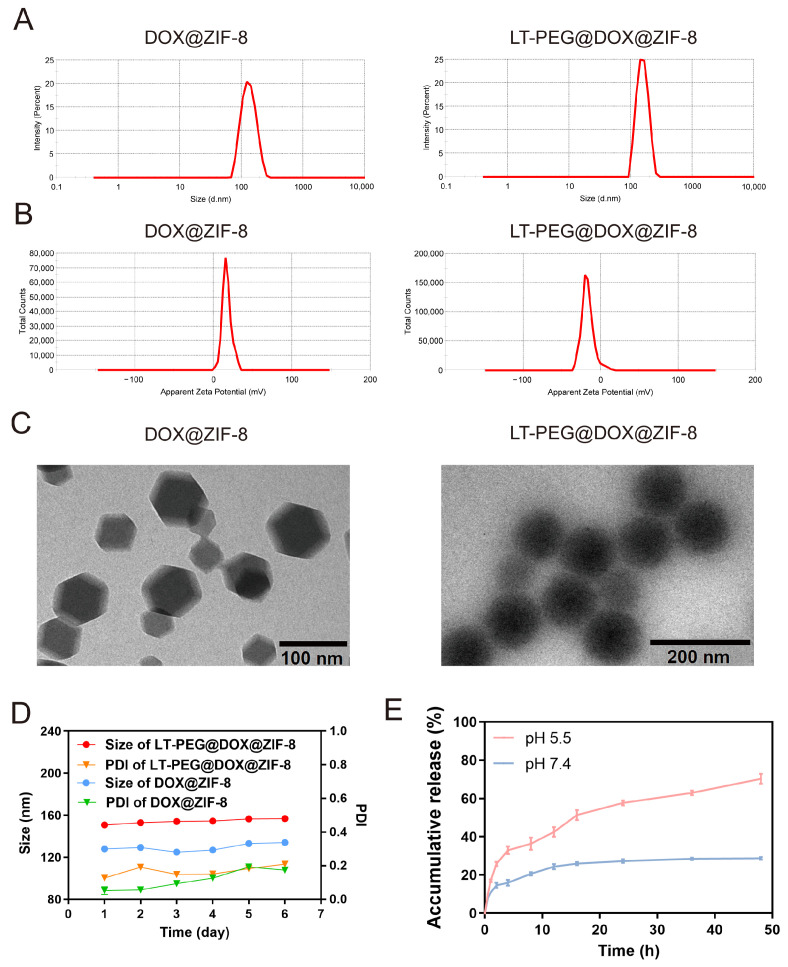
Characterizations of LT-PEG@DOX@ZIF-8. Particle size (**A**), zeta potential (**B**), and TEM images (**C**) of DOX@ZIF-8 and LT-PEG@DOX@ZIF-8. (**D**) The storage stability of nanoparticles with and without the LT peptide for 6 days in PBS of pH 7.4 at 4 °C. (**E**) The pH-responsive release profiles of LT-PEG@DOX@ZIF NPs in PBS of pH 7.4 and 5.5 (means ± SDs, *n* = 3).

DOX@ZIF-8 was successfully obtained by a simple one-pot method. The LT peptide was then covered on the surface of DOX@ZIF-8 to obtain LT-PEG@DOX@ZIF-8 nanoparticles through the electrostatic interaction between LT-PEG-COOH and Zn^2+^ ions of ZIF-8. To study the effect of the LT peptide on the size and surface potential of the nanoparticles, DLS was employed to measure the hydrated particle size and zeta potential of the nanoparticles. It could be seen that the average particle sizes of DOX@ZIF-8 and LT-PEG@DOX@ZIF-8 were 124.8 nm and 152.8 nm, respectively (Figure 2A). Compared with DOX@ZIF-8, the hydrated particle size of LT-PEG@DOX@ZIF-8 slightly increased, probably because LT-PEG covered the surface of the nanoparticles. Meanwhile, as shown in Figure 2B, the surface zeta potentials of DOX@ZIF-8 and LT-PEG@DOX@ZIF-8 were 16.7 mV and −17.0 mV. Obviously, the surface zeta potential of the LT-modified nanoparticles was significantly different from that of the unmodified nanoparticles, with the LT modification changing the nanoparticle surface potential from negative to positive. The surface potential of DOX@ZIF-8 was positive on account of the presence of Zn^2+^ ions. When the nanoparticles were covered with negatively charged LT-PEG, the surface potential was changed from positive to negative, which further proved the successful modification of the LT peptide. Moreover, the TEM images (Figure 2C) demonstrated that the DOX@ZIF-8 NPs presented a homogeneous dodecahedral morphology with sharp edges. In contrast, the LT-PEG@DOX@ZIF-8 NPs exhibited spherical shapes and blurred edges, which were attributed to the successful coverage of LT-PEG on the nanoparticle surface.

The EE% and DL% of LT-PEG@DOX@ZIF-8 were 51.1 ± 0.4% and 8.2 ± 0.4%, which were determined by HPLC. In addition, we investigated the stability of the DOX@ZIF-8 and LT-PEG@DOX@ZIF-8 NPs after storage in PBS (pH 7.4) at 4 °C for 6 days. Both the average particle size and PDI showed negligible change, indicating good stability (Figure 2D). Many studies have reported that ZIF-8 features pH-responsive degradation. To explore the pH-responsive drug release behavior of the LT-PEG@DOX@ZIF-8 nanoparticles, release mediums with pH 7.4 and pH 5.5 were utilized to simulate normal physiological environments and tumor nuclear lysosomes/endosomes, respectively. It can be seen in Figure 2E that the LT-PEG@DOX@ZIF-8 nanoparticles were released rapidly at pH 5.5, with a cumulative release of approximately 70% within 48 h. On the contrary, in the release media at pH 7.4, the release of nanoparticles reached equilibrium at 12 h, and less than 30% was released cumulatively within 48 h. Accordingly, LT-PEG@DOX@ZIF-8 nanoparticles can remain stable under a normal physiological environment, preventing the drug from prematurely leaking and causing damage to normal tissues or organs, while DOX can be rapidly released within tumor cells to exert therapeutic effects, resulting in targeted and controlled release of the loaded drug.

### 3.3. Cellular Uptake Study

**Figure 3:**
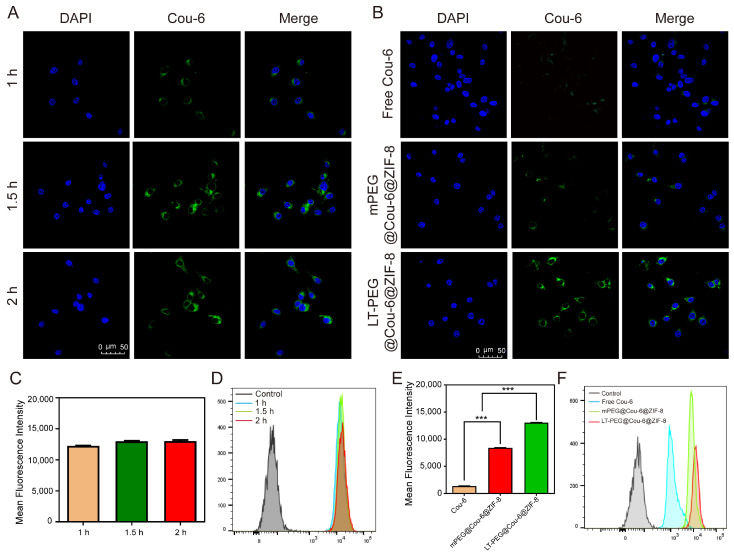
Cellular uptake of LT-PEG@Cou-6@ZIF-8 by C26 cells. (**A**) Confocal images of cellular uptake at 1 h, 1.5 h, and 2 h. (**B**) Confocal images of cellular uptake of different preparations at 1.5 h. Green: Cou-6; blue: DAPI (nuclei). Analysis of cellular uptake at 1 h, 1.5 h, and 2 h using mean fluorescence intensity (**C**) and flow cytometry (**D**). Analysis of cellular uptake of different preparations using mean fluorescence intensity (**E**) and flow cytometry (**F**) (means ± SDs, *n* = 3), *** *p* < 0.001.

Effective cellular uptake is essential for exerting the anti-tumor efficacy of drugs. Herein, coumarin 6 (Cou-6) was adopted in place of DOX encapsulated in ZIF-8 to explore the uptake by C26 cells (Figure S1). Figure 3A presents cellular uptake images of LT-PEG@Cou-6@ZIF-8 at different times, where blue represents the nucleus stained by DAPI and green represents the signal of Cou-6. The images show that the uptake of LT-PEG@Cou-6@ZIF-8 nanoparticles by the C26 cells was rapid, and the fluorescence intensity reached a strong level after 1.5 h of incubation. With the prolonged incubation time, the fluorescence intensity also progressively turned stronger. The results of the quantitative analysis performed by the flow cytometry assay were also similar to those derived by CLSM. In Figure 3C,D, it can be seen that the uptake of nanoparticles by the C26 cells was essentially saturated after incubating for 1.5 h, and longer incubation times brought only slight improvement. Therefore, 1.5 h of incubation was chosen to compare the difference in cellular uptake between the various preparations in the subsequent experiments.

Taking into account the above results, 1.5 h of incubation was set to investigate the cellular uptake by the C26 cells of different preparations. In order to eliminate the interference of PEG, three groups were set up as follows: free Cou-6, mPEG@Cou-6@ZIF-8, and LT-PEG@Cou-6@ZIF-8. According to Figure 3B, the green fluorescent signal was hardly seen in the free Cou-6 group, which means that almost no Cou-6 was taken up by the cells. Similarly, there was only an insignificant amount of the green fluorescent signal in the mPEG@Cou-6@ZIF-8 group, indicating that only low levels of Cou-6 were taken up. On the contrary, extensive green fluorescence signals could be clearly observed in the LT-PEG@Cou-6@ZIF-8 group, suggesting that the cellular uptake efficiency was significantly increased after the modification of LT. Moreover, the quantitative results of the flow cytometry assay were more intuitive. LT-PEG@Cou-6@ZIF-8 displayed the highest cellular uptake efficiency, which was about 9.7-fold higher than free Cou-6 and 1.5-fold higher than mPEG@Cou-6@ZIF-8 (Figure 3E,F). All results indicated that the modification of LT improved the uptake of nanoparticles by the C26 cells.

### 3.4. Cell Viability Assay

**Figure 4:**
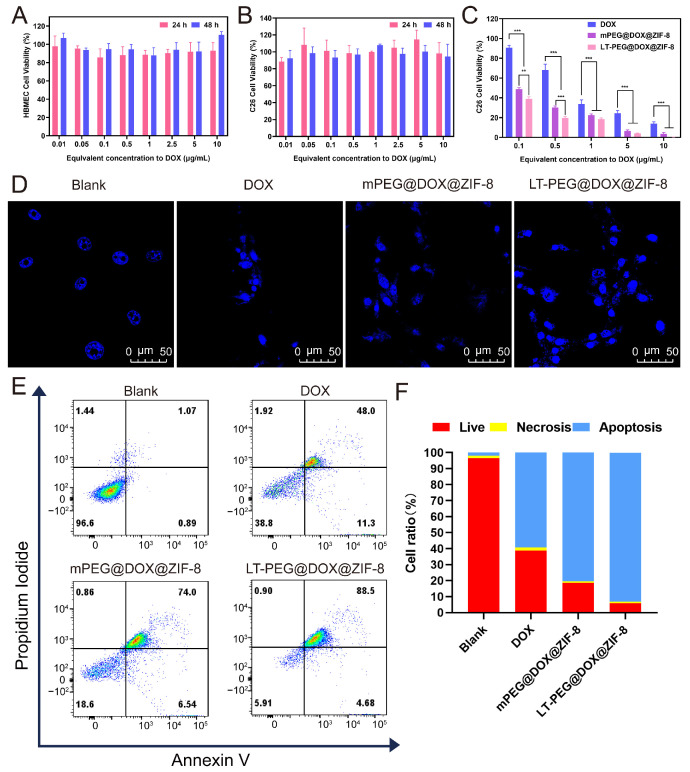
In vitro cytotoxicity and cell apoptosis assays. (**A**) In vitro cell toxicity of blank ZIF-8 on HBMEC cells. (**B**) In vitro cell toxicity of blank ZIF-8 on C26 cells. (**C**) Inhibitory capacity of different preparations against C26 cell proliferation after incubating for 48 h. (**D**) Hoechst staining with different preparations. The apoptosis assay of C26 cells analyzed using flow cytometry (**E**) and quantitative analysis (**F**). Means ± SDs, ** *p* < 0.01, *** *p* < 0.001.

After confirming that LT-PEG@DOX@ZIF-8 nanoparticles could enhance the uptake efficiency in C26 cells, we proceeded to evaluate the in vitro toxicity by the CCK-8 assay. Firstly, the toxicity of a blank carrier material to C26 cells was evaluated. In Figure 4A,B, it can be seen that blank ZIF-8 showed no significant toxicity after incubation with HBMEC and C26 cells for both 24 h and 48 h, with high cell survival rates, all above 85% at the tested concentrations. Therefore, blank ZIF-8 is of low toxicity and has good biocompatibility. Subsequently, the effect of different preparations on the inhibition of C26 cell proliferation was investigated. After incubation for 48 h, all preparations exhibited dose-dependent effects of inhibition on C26 cell proliferation (Figure 4C). The IC_50_ values of mPEG@DOX@ZIF-8 and LT-PEG@DOX@ZIF-8 were 0.075 μg/mL and 0.019 μg/mL, which were remarkably lower than DOX (0.86 μg/mL), suggesting that DOX encapsulated in ZIF-8 greatly enhanced the toxicity and inhibitory ability against tumor cells. Compared to the β-GluNPs/DOX nanoparticles prepared by Rajabi et al. [31], the IC50 of LT-PEG@DOX@ZIF-8 was much lower than that of the β-GluNPs/DOX nanoparticles (0.35 μM), indicating its superiority. Moreover, after the modification of ZIF-8 with LT-PEG-COOH, a significant difference appeared between the toxicity of LT-PEG@DOX@ZIF-8 and mPEG@DOX@ZIF-8 on the C26 cells at a dose close to the IC_50_. However, when the administered concentration was more than 1 μg/mL, the high concentration of DOX suppressed the C26 cells to a lower level, and thus, the difference between the two was not significant. The improved anti-proliferative capacity of LT-PEG@DOX@ZIF-8 may result from more cellular internalization, as evidenced by the cellular uptake assays. Overall, it can be concluded that LT-PEG@DOX@ZIF-8 has a stronger proliferation inhibitory effect on C26 cells than mPEG@DOX@ZIF-8 and free DOX.

### 3.5. In Vitro Cell Apoptosis Study 

Hoechst 33258 is a blue fluorescent dye that can bind to nuclear DNA. When the nuclei of C26 cells with normal morphology and structure were stained with Hoechst 33258, it could be observed that the nuclei were homogeneous blue with an oval shape under CLSM. On the other hand, when the nuclei of the apoptotic C26 cells were stained with Hoechst 33258, it was observed that the nuclear pores were enlarged and the chromatin was condensed, appearing as bright blue with dense fragments. In Figure 4D, it can be observed that the normal cells were regularly round or oval in the blank group, but after treating with DOX, the cells underwent typical apoptosis-like changes, and the nuclei of the cells became dense, thick-stained, or fragmented, with dense blue fluorescence, which indicated that DOX induced apoptosis in the C26 cells. Furthermore, LT-PEG@DOX@ZIF-8 induced the most cell apoptosis compared to the other treatments, as evidenced by the image. Subsequently, similar results were obtained for further quantitative analysis of apoptosis. According to the methods reported in the extensive references [32,33,34,35,36], we adopted Annexin-V/PI double staining to quantitatively examine the induction of apoptosis in the C26 cells by different preparations encapsulated with DOX. The apoptosis rates of the free DOX, mPEG@DOX@ZIF-8, and LT-PEG@DOX@ZIF-8 groups were 59.3%, 80.54%, and 93.18%, respectively (Figure 4E,F). The results of Annexin-V/PI double staining and Hoechst staining corroborated each other, together indicating that LT-PEG@DOX@ZIF-8 NPs have a strong apoptosis-inducing potential. The results of apoptosis showed a similar trend in the inhibition of cell proliferation and were in agreement with the results of CLSM, which further indicated that LT-PEG@DOX@ZIF-8 nanoparticles were more readily taken up by the C26 cells, suggesting that LT-PEG@DOX@ZIF-8 has a strong inhibitory effect on cell proliferation and an enhanced apoptosis-inducing effect on C26 cells.

### 3.6. Wound-Healing Assay

**Figure 5:**
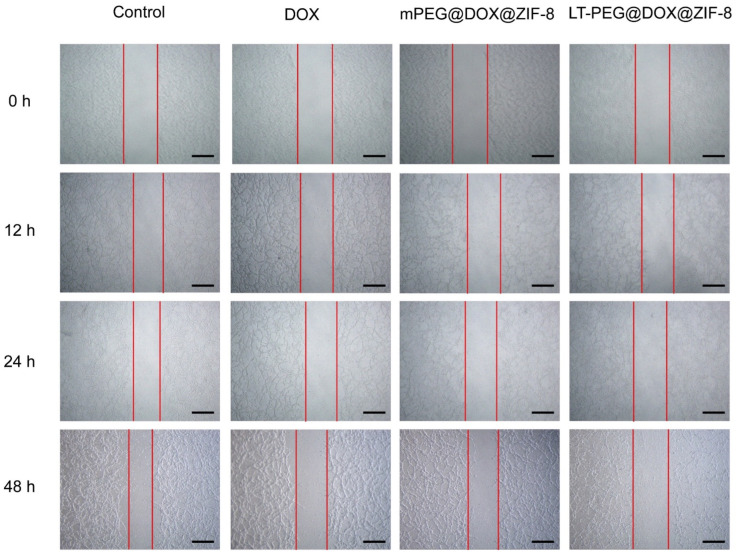
Wound-healing assay of different preparations on C26 cells. Scale bar = 100 μm.

Wound-healing assays were conducted to investigate the effect of different preparations on the migration and invasion of tumor cells. In Figure 5, it can be observed that the scratch area in the control group gradually shrunk, which was driven by the proliferation and movement of the C26 cells over time. Conversely, the scratched areas of the other groups were still clearly visible, suggesting that DOX efficiently inhibited the migration of C26 cells. Furthermore, LT-PEG@DOX@ZIF-8 displayed a stronger resistance to migration than the other groups, probably due to the enhanced cytotoxicity. All of the above results indicated that LT-PEG@DOX@ZIF-8 possessed excellent anti-tumor migration and invasion ability.

### 3.7. In Vivo Biodistribution

**Figure 6:**
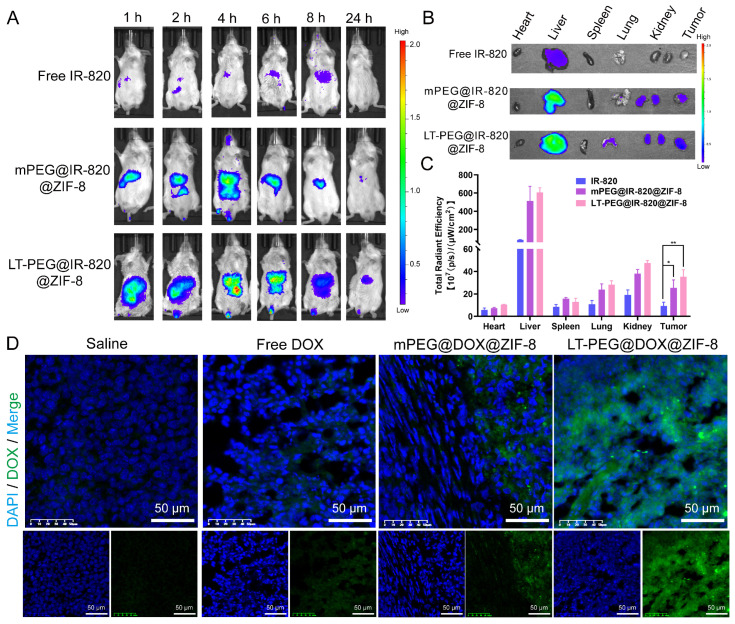
In vivo biodistribution. (**A**) In vivo imaging of mice treated with different preparations encapsulated IR-820 at 1, 2, 4, 6, 8, and 24 h. (**B**) Ex vivo images and (**C**) quantitative analysis of the fluorescence intensities of main organs at 24 h after injection. (**D**) The distribution of DOX in a tumor at 4 h after systemic administration of various preparations. Blue, cell nuclei stained with DAPI; green, DOX. Means ± SDs, * *p* < 0.05, ** *p* < 0.01.

The in vitro cellular uptake assays and proliferation inhibition and apoptosis assays demonstrated that LT-PEG@DOX@ZIF-8 could be effectively adopted and exert anti-tumor effects in C26 cells. However, it is essential for the drug to accumulate effectively in the tumor tissue to be taken up by tumor cells and exert effects in vivo. Thus, we investigated the biodistribution and aggregation of the LT-modified ZIF-8 NPs at tumor sites in vivo after systemic administration. Since the fluorescence signal of DOX could not be captured by the in vivo imaging system, we employed the fluorescent probe instead of DOX to examine the in vivo tumor targeting of LT-modified ZIF-8 nanoparticles. Commonly used in vivo imaging probes in place of model drugs for numerous studies include Dir, ICG, IR-820, Cy 5.5, and Cy5 [37,38,39,40,41,42,43]. Given the similar properties of IR-820 and DOX and the strong fluorescent signal in vivo, we chose IR-820 to encapsulate in ZIF-8 in place of DOX. Mice with colorectal tumors were administrated systemically with various preparations, and then the fluorescence signals were collected at 1, 2, 4, 6, 8, and 24 h using an in vivo imaging system. As can be seen in Figure 6A, free IR-820 displayed substantial accumulation in the liver but almost none in the tumor, and it was metabolized rapidly in vivo. Similarly, mPEG@IR-820@ZIF-8 also presented strong fluorescent intensity in the liver but weak intensity in the tumor within 4 h. Furthermore, the fluorescent signal at the tumor site disappeared at 6 h, indicating that the nanoparticles were rapidly metabolized. In contrast, LT-PEG@IR-820@ZIF-8 consistently demonstrated intense fluorescence signals in the tumor, which reached a maximum at 4 h, signifying the effective accumulation of nanoparticles in the tumor. Meanwhile, the fluorescence signals at the tumor site were still observed at 24 h, implying that LT-modified nanoparticles achieve prolonged retention in the tumor tissue, thus exerting improved efficacy. Subsequently, the mice were euthanized at 24 h, and their major organs (hearts, livers, spleens, lungs, and kidneys) and tumors were removed to measure their fluorescence intensity. The fluorescence signal distribution of ex vivo organs revealed a similar trend to the in vivo imaging, with the LT-PEG@IR-820@ZIF-8 group showing the strongest fluorescence signal in the tumors (Figure 6B). The tumor fluorescence intensity of the mice in the LT-PEG@IR-820@ZIF-8 group was 3.8-fold and 1.3-fold higher than that in the free IR-820 group and the mPEG@IR-820@ZIF-8 group, respectively (Figure 6C). Overall, the LT-modified ZIF-8 nanoparticles exhibited excellent colon cancer targeting ability and allowed prolonged circulation in vivo, which enhanced the accumulation and retention at the tumor site, thus improving the efficacy.

### 3.8. Biodistribution in Tumors

Given that the stability and release behavior of the ZIF-8 NPs encapsulated with IR-820 is different from that of the DOX-encapsulated ZIF-8 NPs, the specific aggregation of LT-PEG@IR-820@ZIF-8 at tumors only demonstrates the tumor-targeting properties of LT-modified ZIF-8 NPs. To further validate the tumor accumulation of ZIF-8 NPs loaded with DOX, different formulations encapsulated with DOX were prepared and investigated for their distribution in tumors. The mice with colorectal tumors were randomly grouped and administered with various preparations via the tail vein. Their tumors were obtained at 4 h post-injection, and frozen sections were prepared to observe the fluorescence signal of DOX by CLSM. In Figure 6D, it is clear that the fluorescence signal of DOX in the tumor tissues of the LT-PEG@DOX@ZIF-8 group showed a remarkably higher level than the free DOX and mPEG@DOX@ZIF-8 groups. The strong fluorescent signal of the LT-PEG@IR-820@ZIF-8 NPs in the tumor, shown by the in vivo imaging system, together with the high distribution of LT-PEG@DOX@ZIF-8 NPs in the tumor frozen sections, allowed us to conclude that LT-PEG@DOX@ZIF-8 NPs could specifically aggregate and target tumors in vivo. All of the above results suggest that LT-PEG@DOX@ZIF-8 can be effectively taken up by colorectal tumor cells and release DOX after systemic administration, which is expected to exert excellent anti-tumor effects in vivo.

### 3.9. In Vivo Anti-Tumor and Anti-Metastasis Efficacy

**Figure 7:**
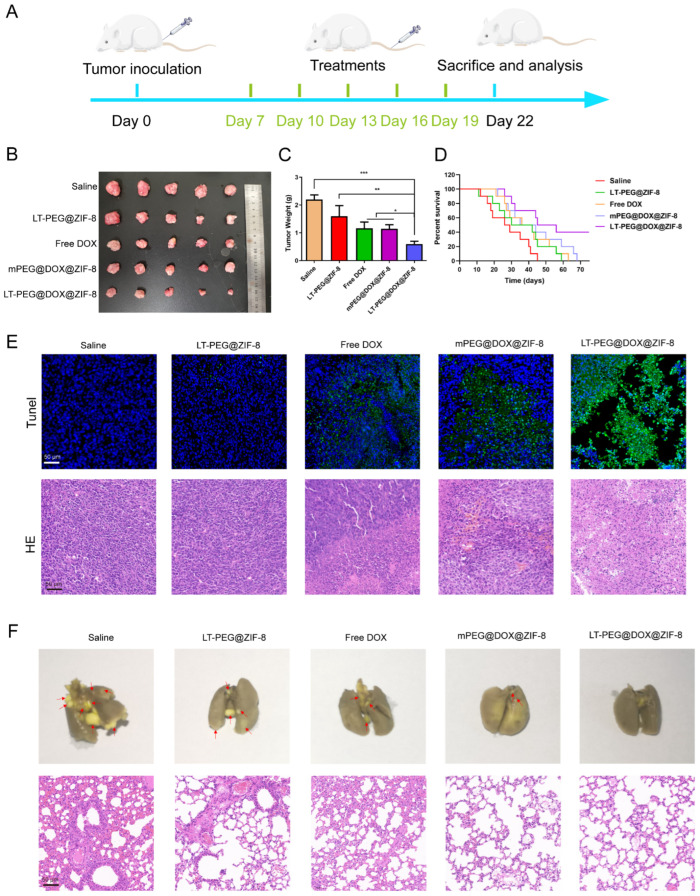
Anti-tumor and antimetastatic efficacy of LT-PEG@DOX@ZIF-8 in vivo. (**A**) Schematic description of treatment against CRC in vivo. (**B**) Photographs of collected colorectal tumors. (**C**) The mean weight of collected colorectal tumors. (**D**) The survival curves of mice given various preparations. (**E**) TUNEL and HE staining sections of tumors from mice treated with various preparations. (**F**) Photographs and HE staining of lungs from mice treated with various preparations. Lung metastases are marked by red arrows. Means ± SDs. * *p* < 0.05, ** *p* < 0.01, *** *p* < 0.001.

Motivated by the enhanced tumor targeting ability, we then explored the anti-tumor effects of LT-PEG@DOX@ZIF-8 in the orthotopic C26 colorectal tumor-bearing mice model. Mice with colorectal tumors were divided randomly into five groups, which were treated with saline, LT-PEG@ZIF-8, free DOX, mPEG@DOX@ZIF-8, and LT-PEG@DOX@ZIF-8 by tail vein injection, respectively. The treatment program followed the schedule shown in Figure 7A. After the last treatment, the mice were executed, and their tumor tissues were harvested and imaged. As can be seen in Figure 7B, free DOX and mPEG@DOX@ZIF-8 exhibited limited therapeutic efficacy, possibly due to rapid clearance or a lack of targetability to accumulate at the tumor site. In contrast, LT-PEG@DOX@ZIF-8 could effectively suppress tumors, as evidenced by the significantly smaller tumor volume compared with other groups. Meanwhile, the measurements of tumor weights for each group also exhibited the same trend (Figure 7C). Due to the specific targeting of LT peptides to colorectal tumors, LT-PEG@DOX@ZIF-8 showed superior tumor suppression than mPEG@DOX@ZIF-8.

The improved tumor ablation ability of LT-PEG@DOX@ZIF-8 inspired us to further explore the improvement in this therapy on the survival of mice with orthotopic colorectal tumors. As can be seen in the survival curves in Figure 7D, the median survival of the mice in the LT-PEG@DOX@ZIF-8 group was prolonged to 50.5 days, longer than other groups with saline (27.5 days), LT-PEG@ZIF-8 (36 days), free DOX (39 days), and mPEG@DOX@ZIF-8 (39.5 days). Compared with Apt-NPs prepared by Jafar Mosafer et al. [44], the survival of the LT-PEG@DOX@ZIF-8-treated mice bearing colorectal tumors was superior to that of Apt-NPs. Hence, it can be concluded that LT-PEG@DOX@ZIF-8 prolonged the survival of the mice with colorectal tumors, which was possibly attributed to its superior anti-tumor effect.

Moreover, HE staining and TUNEL immunostaining were conducted to evaluate the anti-tumor effect and apoptosis against colorectal tumors. In Figure 7E, it can be noted that the tumor tissue in the LT-PEG@DOX@ZIF-8 group demonstrated higher levels of green fluorescent signals than those in other groups in the TUNEL-stained sections, indicating a superior level of apoptosis. Meanwhile, as shown in the HE staining results, the nuclei of the tumor cells were deeper in color in the saline group and the LT-PEG@ZIF-8 group, presenting as dark purple and tightly aligned. The overall color of the LT-PEG@DOX@ZIF-8 group was pink, and the tumor tissues became sparse and possessed the strongest cell destruction and contraction, with the highest density of nuclei absence and apoptosis, which indicated that substantial apoptosis of tumor cells occurred. Such results further prove the potential of LT-PEG@DOX@ZIF-8 for the treatment of colorectal tumors.

Easy lung metastasis is one of the symptoms that occurs in patients suffering from CRC, which is also a hot issue of clinical treatment. Therefore, the lungs of the mice were collected after treatment to study the extent of tumor metastasis to the lungs. As shown in Figure 7F, plenty of metastatic nodules appeared in the lungs of the mice in the saline and LT-PEG@ZIF-8 groups, and there were a few in the free DOX and mPEG@DOX@ZIF-8 groups, whereas almost none were observed in the LT-PEG@DOX@ZIF-8 group. To further analyze the metastatic nodules in the lungs, HE staining was performed for pathological analysis. The results revealed that widespread tumor cells with dense nuclei appeared in the lung tissues of the saline group. Compared with other treatment groups, lung sections of the mice in the LT-PEG@DOX@ZIF-8 group presented normal lung morphology and rarely presented metastatic tumor cells. Thus, LT-PEG@DOX@ZIF-8 NPs have excellent anti-colorectal tumor metastasis to the lung.

### 3.10. In Vivo Biocompatibility and Biosafety Evaluation

**Figure 8:**
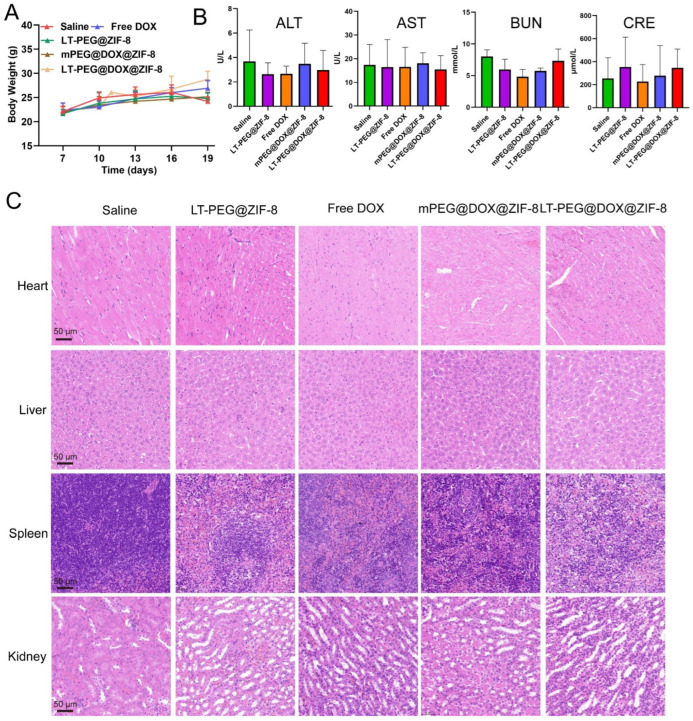
Biosafety evaluation of LT-PEG@DOX@ZIF-8. (**A**) The body weight of mice given various preparations. (*n* = 5). (**B**) ALT, AST, BUN, and CRE levels of mice given various preparations (*n* = 5). (**C**) HE staining images of the main organs of mice given various preparations. Means ± SDs.

Not only is excellent tumor inhibition efficiency required, but high biocompatibility and biosafety are also crucial for the application of LT-PEG@DOX@ZIF-8 in vivo. Therefore, the biosafety of different preparations was assessed during treatment. The changes in the weight of the mice during the treatment are shown in Figure 8A. No obvious change appeared in the weight of the mice in all the groups, but the LT-PEG@DOX@ZIF-8 group of mice experienced the highest degree of elevation in weight. After the treatments, blood was collected from the mice for serum biochemical analyses involving AST, ALT, BUN, and CRE. (Figure 8B). The results revealed that all the serum biochemical indices were not significantly changed between the saline group and others, suggesting that no hepatotoxicity or nephrotoxicity was produced. In addition, to further investigate the damage caused by nanoparticles on the major organs of the mice, we collected the hearts, livers, spleens, and kidneys from the mice in each group for HE staining (Figure 8C). In comparison with the saline group, none of the other treatment groups experienced evident pathological abnormalities, suggesting no apparent toxicity to the main organs. All the results indicated that LT-modified ZIF-8 nanoparticles have good biocompatibility and biosafety.

## 4. Conclusions

In this study, we constructed LT-modified ZIF-8 nanoparticles for the delivery of the chemotherapeutic drug DOX to treat CRC. LT-PEG@DOX@ZIF-8 possessed the optimal particle size and zeta potential, with excellent storage stability, and enabled pH-responsive drug release behavior. The in vitro assays showed that LT-PEG@DOX@ZIF-8 could be efficiently taken up by C26 cells and effectively inhibit the proliferation and induce apoptosis of tumor cells. Moreover, LT-PEG@DOX@ZIF-8 could specifically distribute in colorectal tumors with the remarkable capability to promote tumor ablation in vivo and prolong the survival of mice. Furthermore, LT-PEG@DOX@ZIF-8 effectively inhibited pulmonary metastasis of colorectal tumors with no obvious damage to other major organs, featuring good biocompatibility and biosafety. In conclusion, LT-PEG@DOX@ZIF-8 has great potential for the targeted treatment of CRC.

## Data Availability

All available data are reported in this article.

## Supplementary Materials

The following supporting information can be downloaded at https://www.mdpi.com/article/10.3390/pharmaceutics17020246/s1: Figure S1. Size distribution and surface zeta potential of mPEG@Cou-6@ZIF-8 and LT-PEG@Cou-6@ZIF-8.
