# Supplementary material for: Novel Peptide-Modified Zeolitic Imidazolate Framework-8 Nanoparticles with pH-Sensitive Release of Doxorubicin for Targeted Treatment of Colorectal Cancer
Source: Pharmaceutics. 2025 Feb 13;17(2):246. doi: 10.3390/pharmaceutics17020246 (PMC11858906; doi:10.3390/pharmaceutics17020246)
Supplement: Supplementary file 1 [file pharmaceutics-17-00246-s001.zip › pharmaceutics-3383528-supplementary.pdf]

Novel Peptide-Modified Zeolitic Imidazolate Framework-8 Nanoparticles with pH-Sensitive Release of Doxorubicin for Targeted Treatment of Colorectal Cancer

Liming Gong [1,2,†], Heming Zhao [1,2,†], Liqing Chen [1,2], Yanhong Liu [1,2], Hao Wu [1,2], Chao Liu [1,2], Jing Feng [1,2], Chenfei Liu [1,2], Congcong Xiao [1,2], Qiming Wang [1,2], Mingji Jin [1,2], Zhonggao Gao [1,2], Wei Huang [1,2,] * and Youyan Guan [3,] *

[1] State Key Laboratory of Bioactive Substance and Function of Natural Medicines, Institute of Materia Medica, Chinese Academy of Medical Sciences and Peking Union Medical College, Beijing 100050, China; dawngong@163.com (L.G.); heming.zhao@btyy.com (H.Z.); chenliqing@imm.ac.cn (L.C.); liuyanhong@imm.ac.cn (Y.L.); wuhao931230@163.com (H.W.); liuchao@bme.pumc.edu.cn (C.L.); fengjinga@imm.ac.cn (J.F.); l iuchenfei@imm.ac.cn (C.L.); xiaocongcong@imm.ac.cn (C.X.); wqmxinyou@imm.ac.cn (Q.W.); jinmingji@imm.ac.cn (M.J.); zggao@imm.ac.cn (Z.G.)

[2] Beijing Key Laboratory of Drug Delivery Technology and Novel Formulations, Department of Pharmaceutics, Institute of Materia Medica, Chinese Academy of Medical Sciences and Peking Union Medical College, Beijing 100050, China

[3] Department of Urology, National Cancer Center, National Clinical Research Center for Cancer, Cancer Hospital, Chinese Academy of Medical Sciences and Peking Union Medical College, Beijing 100021, China

* Correspondence: huangwei@imm.ac.cn (W.H.); guanyouyan@cicams.ac.cn (Y.G.)

[†] These authors contributed equally to this work.

^{1}H nuclear magnetic resonance (NMR) spectroscopy (500 MHz) acquisition conditions: Solvent: D_2O; Temperature:25 °C; Proton; Pulse program: zg 30; TD = 65536; DS = 2; NS = 16; TD0 = 1; D1 = 1.

Supporting figures:

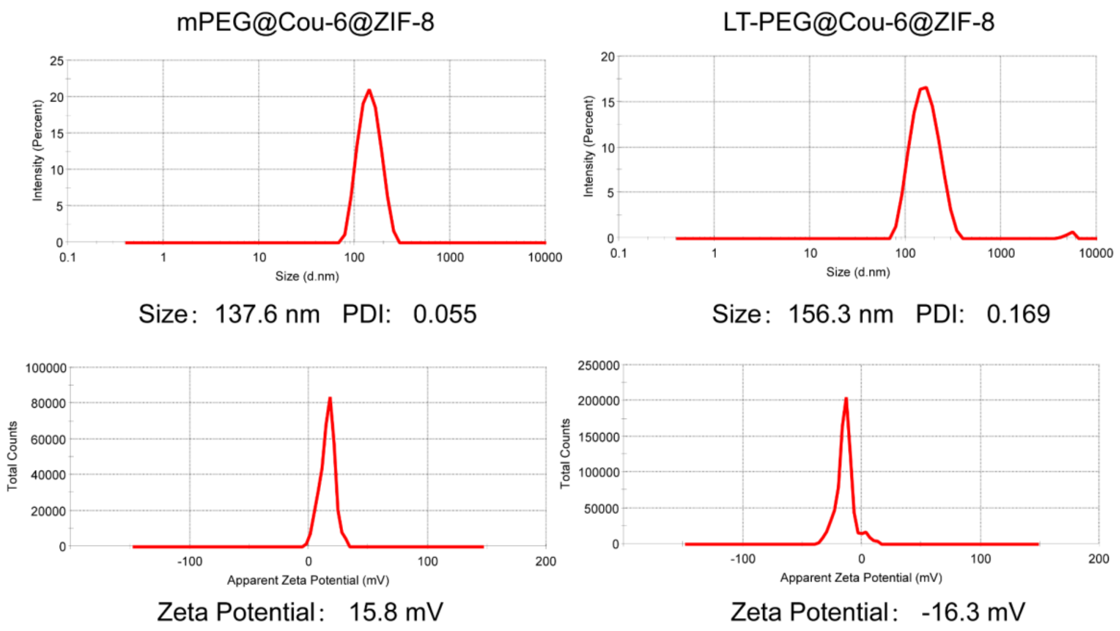

Figure S1. Size distribution and surface zeta potential of mPEG@Cou-6@ZIF-8 and LT-PEG@Cou-6@ZIF-8.
